# Approximation of the multiplicatives on random multi-normed space

**DOI:** 10.1186/s13660-017-1478-9

**Published:** 2017-08-31

**Authors:** Ravi P Agarwal, Reza Saadati, Ali Salamati

**Affiliations:** 1 Department of Mathematics, Texas A & M University - Kingsville, Kingsville, TX 78363 USA; 2 Department of Mathematics, Iran University of Science and Technology, Tehran, Iran

**Keywords:** 39A10, 39B52, 39B72, 46L05, 47H10, 46B03, 54E40, 54E35, 54H25, approximation, homomorphisms, random multi-normed space, dual random multi-normed space

## Abstract

In this paper, we consider random multi-normed spaces introduced by Dales and Polyakov (Multi-Normed Spaces, 2012). Next, by the fixed point method, we approximate the multiplicatives on these spaces.

## Introduction

The concept of random normed spaces and their properties are discussed in [2]. Also, the concept of multi-normed spaces was introduced by Dales and Polyakov. In this paper we combine the mentioned concepts and introduce random multi-normed spaces. Next, we get an approximation for homomorphisms in these spaces. For more results and applications, one can see [3–23].

### Definition 1.1

Let $(E,\mu,\ast)$ be a random normed space. ∗ is a continuous t-norm. A multi-random norm on $\{ E^{k},k\in\mathbb{N} \} $ is sequence $\{N_{k}\} $ such that $N_{k} $ is a random norm on $E^{k}$ ($k\in\mathbb{N}$), $\mu_{x}^{1}(t)= \mu_{x}(t)$ for each $x\in E$ and $t\in\mathbb{R}$ and the following axioms are satisfied for each $k\in\mathbb{N}$ with $k\geq2$:

1. $\mu_{A_{\sigma}(x)}^{k}(t)=\mu_{x}^{k}(t)$, for each $\sigma\in\sigma_{k}$, $x\in E^{k}$, $t\in\mathbb{R}$,
2. $\mu_{M_{\alpha}(x)}^{k}(t)\geq \mu_{\max_{i\in\mathbb{N}_{k}} \vert \alpha_{i} \vert x}^{k}(t)$, for each $\alpha= (\alpha_{1},\ldots,\alpha_{k})\in\mathbb{R}^{k}$, $x\in E^{k}$, $t\in\mathbb{R}$,
3. $\mu_{(x_{1},\ldots,x_{k},0)}^{k+1}(t)=\mu_{(x_{1},\ldots,x_{k})} ^{k}(t)$, for each $x_{1},\ldots,x_{k}\in E$ and $t\in\mathbb{R}$,
4. $\mu_{(x_{1},\ldots,x_{k},x_{k})}^{k+1}(t)=\mu_{(x_{1},\ldots,x _{k})}^{k}(t)$, for each $x_{1},\ldots,x_{k}\in E$ and $t\in\mathbb{R}$.

In this case $\{(E^{k},\mu^{k},\ast),k\in\mathbb{N} \}$ is called a random multi-normed space. Moreover, if axiom (NF4) is replaced by the following axiom:

(DF4) $\mu_{(x_{1},\ldots,x_{k},x_{k})}^{k+1}(t)=\mu_{(x_{1},\ldots,2x _{k})}^{k}(t)$, for each $x_{1},\ldots,x_{k}\in E$ and $t\in\mathbb{R}$,

then $\{\mu^{k} \}$ is called a dual random multi-normed and $\{(E ^{k},\mu^{k},\ast), k\in\mathbb{N} \}$ is called a dual random multi-normed space.

## Approximation of the multiplicatives

We apply fixed point theory [24] to get an approximation for multiplicatives. A metric *d* on non-empty set ϒ with range $[0,\infty]$ is called a generalized metric.

### Lemma 2.1

[25, 26]

*Let* $k\in\mathbb{N}$, *and let* *E* *and* *F* *be linear spaces such that* $(F^{k},\mu^{k},*)$ *is a complete random multi*-*normed space*. *Let there exist* $0\leq M<1$, $\lambda> 0$, *and a function* $\psi: E^{k} \longrightarrow[0,\infty)$ *such that*

2.1 $$\begin{aligned} \psi(\lambda x_{1},\ldots,\lambda x_{k})\leq \lambda M\psi (x_{1},\ldots,x _{k}) \quad(x_{1}, \ldots,x_{k}\in E). \end{aligned}$$

*We set* $\Upsilon:= \{ \eta:E \longrightarrow F:\eta(0)=0 \}$, *and define* $d:\Upsilon\times\Upsilon$ *on* $[0,\infty]$ *by*

$$\begin{aligned}& d(\eta,\zeta) \\& \quad = \inf\biggl\{ c> 0: \mu_{(\eta(x_{1})-\zeta(x_{1}),\ldots,\eta(x_{k})-\zeta(x_{k}))}(ct) \geq\frac{t}{t+\psi(x_{1},\ldots,x_{k})}, x_{1},\ldots,x_{k} \in E \biggr\} . \end{aligned}$$

*Then* $(\Upsilon,d)$ *is a complete generalized metric space*, *and the mapping* $J: \Upsilon\longrightarrow\Upsilon$ *defined by* $(Jg)(x):=\frac{g( \lambda x)}{\lambda}$ ($x\in\Upsilon$) *is a strictly contractive mapping*.

### Theorem 2.2

*Let* *E* *be a linear space and let* $((F^{n},\mu ^{n},*):n\in\mathbb{N})$ *be a complete random multi*-*normed space*. *Let* $k\in\mathbb{N}$ *and let there exist* $0\leq M_{0} < 1$ *and a function* $\varphi:E^{2k} \longrightarrow [0,\infty)$ *satisfying*

2.2 $$ \varphi(2x_{1},2y_{1},\ldots,2x_{k},2y_{k}) \leq2M_{0}\varphi(x_{1},y _{1}, \ldots,x_{k},y_{k}) $$

*for all* $x_{1},y_{1},\ldots,x_{k},y_{k}\in E$. *Suppose that* $f:E \longrightarrow F$ *is a mapping with* $f(0)=0$ *and*

2.3 $$\begin{aligned}& \mu^{k}_{(f(\lambda x_{1}+\lambda y_{1})-\lambda f(x_{1})-\lambda f(y _{1}),\ldots,f(\lambda x_{k}+\lambda y_{k})-\lambda f(x_{k})-\lambda f(y _{k}))}(t) \\& \quad \geq \frac{t}{t+\varphi(x_{1},y_{1},\ldots,x_{k},y_{k})}, \end{aligned}$$

2.4 $$\begin{aligned}& \mu^{k}_{(f(x_{1}y_{1})-f(x_{1})f(y_{1}),\ldots ,f(x_{k}y_{k})-f(x_{k})f(y _{k}))}(t)\geq\frac{t}{t+\varphi(x_{1},y_{1},\ldots,x_{k},y_{k})}, \end{aligned}$$

*for all* $\lambda\in\mathbb{T}:=\{\lambda\in\mathbb{C}: \vert \lambda \vert =1\}$ *and* $x_{1},y_{1},\ldots,x_{k},y_{k}\in E$, $t> 0$.

*Then*

2.5 $$ H(x):=\lim_{n\rightarrow\infty}2^{n}f\biggl( \frac{x}{2^{n}}\biggr) $$

*exists for any* $x_{1},\ldots,x_{k} \in E$ *and defines a random homomorphism* $H:E \longrightarrow F$ *such that*

2.6 $$\begin{aligned}& \mu_{(f(x_{1})-H(x_{1}),\ldots,f(x_{k})-H(x_{k}))}(t) \geq \frac{(1-M _{0})t}{(1-M_{0})t+M_{0}\psi(x_{1},\ldots,x_{k})}, \end{aligned}$$

2.7 $$\begin{aligned}& \psi(x_{1},\ldots,x_{k})= \varphi\biggl( \frac{x_{1}}{2},\frac {x_{1}}{2},\ldots,\frac{x _{k}}{2},\frac{x_{k}}{2} \biggr), \end{aligned}$$

*for all* $x_{1},\ldots,x_{k} \in E$ *and* $t> 0$.

### Proof

Let $x_{1}=\frac{x_{1}}{2},\ldots,x_{k}=\frac{x_{k}}{2}$, $y_{1}=\frac{y _{1}}{2},\ldots,y_{k}=\frac{y_{k}}{2}$ in (2.2). We get

2.8 $$ \varphi(x_{1},y_{1},\ldots,x_{k},y_{k}) \leq2M_{0}\varphi\biggl( \frac{x_{1}}{2},\frac{y_{1}}{2},\ldots, \frac{x_{k}}{2},\frac{y_{k}}{2}\biggr), $$

since *f* is odd, $f(0)=0$. So $\mu_{f(0)}(\frac{t}{2})=1$. Letting $\lambda=1 $ and $y= x$, we get

2.9 $$ \mu^{k}_{(f(2x_{1})-2f(x_{1}),\ldots,f(2x_{k})-2f(x_{k}))}(t) \geq\frac{t}{t+ \varphi(x_{1},x_{1},\ldots,x_{k},x_{k})} $$

for all $x_{1},y_{1},\ldots,x_{k},y_{k}\in E$. Consider the following set:

$$ s=:\{g:E \longrightarrow F \} $$

and introduce the generalized metric on *s*:

$$\begin{aligned}& d(g,h) \\& \quad= \inf\biggl\{ \nu\in\mathbb{R}_{+} :\mu^{k}_{(g(x_{1})-h(x_{1}),\ldots,g(x _{k})-h(x_{k}))}( \nu t)\geq\frac{t}{t+\varphi(x_{1},\ldots,x_{k})}, x _{1},\ldots,x_{k}\in E,t> 0 \biggr\} , \end{aligned}$$

where, as usual, $\inf\phi=+\infty$. It is easy to show $(s,d)$ is complete. Now, we consider the linear mapping $J:s \longrightarrow s$ such that

$$ J\bigl(g(x)\bigr) :=2g \biggl( \frac{x}{2} \biggr) $$

for all $x \in E$. Let $g,h \in s$ be given such that $d(g,h)=\varepsilon $. Then we have

$$ \mu^{k}_{(g(x_{1})-h(x_{1}),\ldots,g(x_{k})-h(x_{k}))}(\varepsilon t) \geq\frac{t}{t+\varphi(x_{1},x_{1},\ldots,x_{k},x_{k})}, $$

for all $x_{1},\ldots,x_{k} \in E$ and all $t> 0$ and hence we have

$$\begin{aligned} \mu^{k}_{(Jg(x_{1})-Jh(x_{1}),\ldots,Jg(x_{k})-Jh(x_{k}))} ( M_{0} \varepsilon t ) &=\mu^{k}_{2g(\frac{x_{1}}{2})-2h(\frac{x_{1}}{2}),\ldots,2g( \frac{x_{k}}{2})-2h(\frac{x_{k}}{2}))} ( M_{0}\varepsilon t ) \\ &=\mu^{k}_{g(\frac{x_{1}}{2})-h(\frac{x_{1}}{2}),\ldots,g( \frac{x_{k}}{2})-h(\frac{x_{k}}{2}))} \biggl( \frac{M_{0}}{2} \varepsilon t \biggr) \\ &\geq\frac{\frac{M_{0}}{2}t}{\frac{M_{0}}{2}+\varphi ( \frac{x _{1}}{2},\frac{x_{1}}{2},\ldots,\frac{x_{k}}{2},\frac{x_{k}}{2} ) } \\ &\geq\frac{\frac{M_{0}}{2}t}{\frac{M_{0}}{2}+\frac{M_{0}}{2}\varphi (x_{1},x_{1},\ldots,x_{k},x_{k})} \\ &=\frac{t}{t+\varphi(x_{1},x_{1},\ldots,x_{k},x_{k})} \end{aligned}$$

for all $x_{1},\ldots,x_{k} \in E$ and $t> 0$. Then $d(g,h)=\varepsilon $ implies that $d(J{g},J{h})\leq M_{0}\varepsilon$. This means that

$$ d(J{g},J{h})\leq M_{0}\varepsilon $$

for all $g,h\in s$. It follows that

$$ \mu_{(f(x_{1})-2f(\frac{x_{1}}{2}),\ldots,f(x_{k})-2f(\frac{x_{k}}{2}))} \biggl( \frac{M_{0}}{2}t \biggr) \geq\frac{t}{t+\varphi (x_{1},x_{1},\ldots,x _{k},x_{k})} $$

for all $x_{1},\ldots,x_{k} \in E$ and $t> 0$. So $d(f,J{f})\leq\frac{M _{0}}{2}$.

Now, there exists a mapping $H: E \longrightarrow F$ satisfying the following:

1. *H* is a fixed point of *J*, *i.e.*,
  2.10 $$ H \biggl( \frac{x}{2} \biggr) =\frac{1}{2}H(x) $$
  for all $x \in E$. Since $f:E \longrightarrow E$ is odd, $H:E \longrightarrow F$ is an odd mapping. The mapping *H* is a unique fixed point of *J* in the set
  $$ M=\bigl\{ g\in s:d(f,g)< \infty\bigr\} . $$
  This implies that *H* is a unique mapping satisfying (2.10) such that there exists a $\nu\in(0,\infty)$ satisfying
  $$ \mu^{k}_{(f(x_{1})-H(x_{1}),\ldots,f(x_{k})-H(x_{k}))}(\nu t)\geq\frac{t}{t+ \varphi(x_{1},\ldots,x_{k})} $$
  for all $x_{1},\ldots,x_{k} \in E$,
2. $d(J^{n}f,H)\rightarrow0 $ as $n\rightarrow\infty$. This implies that
  $$ \lim_{n\rightarrow\infty}2^{n}f \biggl( \frac{x}{2_{n}} \biggr) =H(x) $$
  for all $x \in E$,
3. $d(f,H)\leq\frac{1}{1-M_{0}}d(f,Jf)$, which implies
  $$ d(f,H)\leq\frac{M_{0}}{2-2M_{0}}. $$

Put $\lambda=1$ in (2.3). Then

$$\begin{aligned}& \mu^{k}_{(2^{n}(f(\frac{x_{1}}{2^{n}}+\frac{y_{1}}{2^{n}})-f(\frac{x _{1}}{2^{n}})-f(\frac{y_{1}}{2^{n}})),\ldots,2^{n}(f(\frac {x_{k}}{2^{n}}+\frac{y _{k}}{2^{n}})-f(\frac{x_{k}}{2^{n}})-f(\frac{y_{k}}{2^{n}})))}(t) \\& \quad \geq \frac{\frac{t}{2^{n}}}{\frac{t}{2^{n}}+ \frac{M_{0}^{n}}{2^{n}}\varphi(x_{1},y_{1},\ldots,x_{k},y_{k})} \end{aligned}$$

for all $x_{1},\ldots,x_{k},y_{1},\ldots,y_{k} \in E$, $t> 0$ and $n\geq1$. Since

$$ \lim_{n\rightarrow\infty} \frac{\frac{t}{2^{n}}}{\frac{t}{2^{n}}+\frac{M _{0}^{n}}{2^{n}}\varphi(x_{1},y_{1},\ldots,x_{k},y_{k})}=1 $$

for all $x_{1},\ldots,x_{k},y_{1},\ldots,y_{k} \in E$, $t> 0$. It follows that

$$ \mu^{k}_{(H(x_{1}+y_{1})-H(x_{1})-H(y_{1}),\ldots,H(x_{k}+y_{k})-H(x_{k})-H(y _{k}))}(t)= 1 $$

for all $x_{1},\ldots,x_{k},y_{1},\ldots,y_{k} \in E$, $t> 0$. So mapping $H:E \longrightarrow F$ is Cauchy additive.

Let $y_{1}=x_{1},\ldots,y_{k}=x_{k}$ in (2.3). Then we have

$$ \mu^{k}_{2^{n}(f(\frac{\beta x_{1}}{2^{n}})-f( \frac{\beta x_{1}}{2^{n}}),\ldots,f(\frac{\beta x_{k}}{2^{n}})-f(\frac{ \beta x_{k}}{2^{n}}))}\bigl(2^{n}t\bigr)\geq \frac{t}{t+\varphi( \frac{x_{1}}{2^{n}},\frac{x_{1}}{2^{n}},\ldots,\frac{x_{k}}{2^{n}},\frac{x _{k}}{2^{n}})} $$

for all $\lambda,\beta\in\mathbb{T}$, $\lambda=\frac{\beta}{2}$, $x_{1},\ldots,x_{k} \in E$, $t> 0$ and $n\geq1$. So we have

$$ \mu^{k}_{2^{n}(f(\frac{\beta x_{1}}{2^{n}})-f( \frac{\beta x_{1}}{2^{n}}),\ldots,f(\frac{\beta x_{k}}{2^{n}})-f(\frac{ \beta x_{k}}{2^{n}}))}(t)\geq\frac{\frac{t}{2^{n}}}{\frac {t}{2^{n}}+\frac{M _{0}^{n}}{2^{n}}\varphi(x_{1},x_{1},\ldots,x_{k},x_{k})} $$

for all $\beta\in\mathbb{T}$, $x_{1},\ldots,x_{k} \in E$, $t> 0$ and $n\geq1$. We have

$$ \lim_{n\rightarrow\infty}\frac{\frac{t}{2^{n}}}{\frac{t}{2^{n}}+\frac{M _{0}^{n}}{2^{n}}\varphi(x_{1},x_{1},\ldots,x_{k},x_{k})}=1 $$

for all $x_{1},\ldots,x_{k} \in E$, $t> 0$, and

$$ \mu^{k}_{(H(\beta x_{1})-\beta H(x_{1}),\ldots,H(\beta x_{k})-\beta H(x _{k}))}(t)=1 $$

for all $\beta\in\mathbb{T}$, $x_{1},\ldots,x_{k} \in E$, $t> 0$. Thus, the additive mapping $H:E \longrightarrow F$ is $\mathbb{R}$-linear. From (2.4), we have

$$\begin{aligned}& \mu^{k}_{(4^{n}f(\frac{x_{1}}{2^{n}}\frac{y_{1}}{2^{n}})-2^{n}f(\frac{x _{1}}{2^{n}})2^{n}f(\frac{y_{1}}{2^{n}}),\ldots,4^{n} f( \frac{x_{k}}{2^{n}}\frac{y_{k}}{2^{n}})-2^{n}f(\frac {x_{k}}{2^{n}})2^{n}f(\frac{y _{k}}{2^{n}}))}\bigl(4^{n}t\bigr) \\& \quad \geq\frac{t}{t+\varphi(\frac{x_{1}}{2^{n}},\ldots, \frac{x_{k}}{2^{n}},\frac{y_{1}}{2^{n}},\ldots,\frac{y_{k}}{2^{n}})} \end{aligned}$$

for all $x_{1},\ldots,x_{k} \in E$, $t> 0$ and $n\geq1$.

Then we have

$$\begin{aligned}& \mu^{k}_{(4^{n}f(\frac{x_{1}}{2^{n}}\frac{y_{1}}{2^{n}})-2^{n}f(\frac{x _{1}}{2^{n}})2^{n}f(\frac{y_{1}}{2^{n}}),\ldots,4^{n} f( \frac{x_{k}}{2^{n}}\frac{y_{k}}{2^{n}})-2^{n}f(\frac {x_{k}}{2^{n}})2^{n}f(\frac{y _{k}}{2^{n}}))}\bigl(4^{n}t\bigr) \\& \quad \geq\frac{\frac{t}{4^{n}}}{\frac{t}{4^{n}}+\frac{M_{0}^{n}}{t^{n}} \varphi(x_{1},\ldots,x_{k},y_{1},\ldots,y_{k})} \end{aligned}$$

for all $x_{1},\ldots,x_{k} \in E$, $t> 0$ and $n\geq1$.

Since

$$ \lim_{n\rightarrow\infty} \frac{\frac{t}{4^{n}}}{\frac{t}{4^{n}}+\frac{M _{0}^{n}}{t^{n}}\varphi(x_{1},\ldots,x_{k},y_{1},\ldots,y_{k})}=1 $$

for all $x_{1},\ldots,x_{k} \in E$, $t> 0$, we have

$$ \mu^{k}_{(H(x_{1}y_{1})-H(x_{1})H(y_{1}),\ldots,H(x_{k}y_{k})-H(x_{k})H(y _{k}))}(t)=1 $$

for all $x_{1},\ldots,x_{k},y_{1},\ldots,y_{k} \in E$, $t> 0$. Thus, the mapping $H:E \longrightarrow F$ is multiplicative. Therefore, there exists a unique random homomorphism $H:E\longrightarrow F$ satisfying (2.6), and this completes the proof. □

## Approximation in dual random multi-normed space

The following lemma is an immediate result of the definition of random multi-normed space.

### Lemma 3.1

*Let* $\{(E^{k},\mu^{k},\ast),k\in\mathbb{N}\}$ *be a dual random multi*-*normed space*, $k,n\in\mathbb{N}$, $x_{1},x_{2},\ldots ,x_{k},x_{k+1},\ldots,x _{k+n}\in\mathbb{E}$ *and* $\lambda_{1},\ldots,\lambda_{k}$ *be real numbers of absolute value* 1. *Then we have*:

(i) $\mu_{(\lambda_{1}x_{1},\ldots,\lambda_{k}x_{k})}^{k}(t)= \mu_{(x_{1},\ldots,x_{k})}^{k}(t)$,
(ii) $\mu_{(x_{1},\ldots,x_{k})}^{k}(t)\geq \mu_{(x_{1},\ldots,x_{k},x_{k+1})}^{k+1}(t)$,
(iii) $\mu_{(x_{1},\ldots,x_{k},x_{k+1},\ldots,x_{k+n})}^{k+n}(t) \geq T_{M}(\mu_{(x_{1},\ldots,x_{k})}^{k}(\alpha t), \mu_{(x_{k+1},\ldots,x_{k+n})}^{n}(\beta t))$, *where* $\alpha,\beta \geq0$ *and* $\alpha+ \beta=1$,
(iv) $\min_{i\in{\mathbb{N}_{k}}} \mu_{x_{i}}(t)\geq \mu_{(x_{1},\ldots,x_{k})}^{k}(t)\geq\min_{i\in\mathbb{N}_{k}} \mu_{x _{i}}(\alpha_{i}t)$,

*where* $\alpha_{1},\ldots,\alpha_{k} \geq0 $ *and* $\sum_{i=1}^{k}\alpha _{i}=1$. *In particular*, *we have*

$$ \mu_{(x_{1},\ldots,x_{k})}^{k}(t)\geq\min_{i\in\mathbb{N}_{k}} \mu_{kx _{i}}(t). $$

### Theorem 3.2

*Let* *E* *be a linear space*, *and* $\{(E^{k},\mu^{k},\ast),k\in \mathbb{N}\}$ *be a random multi space*. *Let* $\alpha\in(0,1)$ *and* $f:E\longrightarrow F$ *is a mapping satisfying* $f(0)=0$ *and*

3.1 $$ \mu_{ ( f(\frac{x_{1}+y_{1}}{2})-\frac{f(x_{1})}{2}- \frac{f(y_{1})}{2},\ldots,f(\frac{x_{k}+y_{k}}{2})-\frac {f(x_{k})}{2}-\frac{f(y _{k})}{2} ) }^{k} \biggl( \frac{t}{s} \biggr) \geq1-\frac{\alpha}{t}, $$

*where* $x_{1},\ldots,x_{k},y_{1},\ldots,y_{k}\in E$ *and* $t,s\in\mathbb{N}$ *with the greatest common divisor* $(t,s)=1$.

*Then there exists a unique additive mapping* $T:E\longrightarrow F$ *such that*

3.2 $$ \mu_{(f(x_{1})-T(x_{1}),\ldots,f(x_{k})-T(x_{k}))}^{k} \biggl( \frac {2t}{s} \biggr) \geq1-\frac{\alpha}{t} $$

*for all* $x_{1},\ldots,x_{k}\in E$ *and* $t,s\in\mathbb{N}$ *with* $(t,s)=1$.

### Proof

Replacing $x_{1},\ldots,x_{k}$ and $y_{1},\ldots,y_{k}$ by $2x_{1},\ldots,2x _{k}$ and $0,\ldots,0$ in (3.1), respectively, yields

3.3 $$ \mu_{(2f(x_{1})-f(2x_{1}),\ldots,2f(x_{k})-f(2x_{k}))}^{k} \biggl( \frac {2t}{s} \biggr) \geq1-\frac{\alpha}{t}. $$

Replacing $x_{1},\ldots,x_{k}$, *t*, *s* by $2x_{1},\ldots,2x_{k}$, 2*t*, 2*s*, respectively, in (3.3) and repeating this process for n-time ($n\in\mathbb{N}$), it follows that

3.4 $$ \mu_{ ( \frac{f(2^{n-1}x_{1})}{2^{n-1}}- \frac{f(2^{n}x_{1})}{2^{n}},\ldots,\frac{f(2^{n-1}x_{k})}{2^{n-1}}-\frac {f(2^{n}x _{k})}{2^{n}} ) }^{k} \biggl( \frac{t}{2^{n-1}s} \biggr) \geq1-\frac{ \alpha}{2^{n-1}t} $$

for $n,m\in\mathbb{N}$ with $n> m$. Using (3.4) and (RN2) we get

$$ \mu_{ ( \frac{f(2^{m}x_{1})}{2^{m}}-\frac {f(2^{n}x_{1})}{2^{n}},\ldots,\frac{f(2^{m}x _{k})}{2^{m}}-\frac{f(2^{n}x_{k})}{2^{n}} ) }^{k} \Biggl( \sum_{i=m} ^{n-1}2^{-i}\frac{t}{s} \Biggr) \geq1-\frac{\alpha}{2^{m}t}. $$

Then

3.5 $$ \mu_{ ( \frac{f(2^{m}x_{1})}{2^{m}}-\frac {f(2^{n}x_{1})}{2^{n}},\ldots,\frac{f(2^{m}x _{k})}{2^{m}}-\frac{f(2^{n}x_{k})}{2^{n}} ) }^{k} \biggl( \frac {t}{s} \biggr) \geq1-\frac{\alpha}{2^{m}t} $$

for $x\in E$. Then, replacing $x_{1},\ldots,x_{k}$ by $x,2x,\ldots,2^{k-1}x$ in (3.5), we have

3.6 $$ \begin{aligned}[b] & \mu_{ ( \frac{f(2^{m}x)}{2^{m}}-\frac{f(2^{n}x)}{2^{n}},\ldots, \frac{f(2^{m+k-1}x)}{2^{m+k-1}}-\frac{f(2^{n+k-1}x)}{2^{n+k-1}} ) } ^{k} \biggl( \frac{t}{s} \biggr) \\ & \quad \geq 1-\frac{\alpha}{2^{m}t} \\ & \quad \geq 1-\frac{\alpha}{2^{m}}. \end{aligned} $$

Let $\varepsilon> 0$ be given. Then there exists $n_{0} \in \mathbb{N}$ such that $\frac{\alpha}{2^{n_{0}}}< \varepsilon$. Now we substitute *m*, *n* with *n*, $n+p$ ($p\in\mathbb{N}$), respectively, in (3.6), for each $n\geq n_{0}$, and we get

$$\begin{aligned} \mu_{ ( \frac{f(2^{n}x)}{2^{n}}-\frac{f(2^{n+p}x)}{2^{n+p}},\ldots, \frac{f(2^{n+k-1}x)}{2^{n+k-1}}-\frac {f(2^{n+p+k-1}x)}{2^{n+p+k-1}} ) } ^{k} \biggl( \frac{t}{s} \biggr) & \geq 1-\frac{\alpha}{2^{n}t} \\ &> 1-\varepsilon. \end{aligned}$$

By Lemma 3.1, we have

3.7 $$ \mu_{\frac{f(2^{n}x)}{2^{n}}-\frac{f(2^{n+p}x)}{2^{n+p}}} \biggl( \frac {t}{s} \biggr) > 1- \varepsilon $$

for all $n> n_{0}$ and $p\in\mathbb{N}$. The density of rational numbers in $\mathbb{R}$ is useful in checking correctness of (3.6) with positive real number *r* instead of $\frac{t}{s}$. Then we have

$$ \mu_{\frac{f(2^{n}x)}{2^{n}}-\frac{f(2^{n+p}x)}{2^{n+p}}}(r)> 1- \varepsilon $$

for each $x\in E$, $r\in\mathbb{R^{+}}$, $n\geq n_{0}$ and $p\in\mathbb{N}$. Then $\{\frac{f(2^{n}x)}{2^{n}}\}$ is a Cauchy sequence, so it is convergent in the random multi-Banach space $\{(E^{k},\mu^{k},\ast),k\in\mathbb{N}\}$. Setting $T(x):= \lim_{n\rightarrow\infty}\frac{f(2^{n}x)}{2^{n}}$ and applying again Lemma 3.1, for each $r> 0$, we have

$$ \mu_{ ( \frac{f(2^{n}x_{1})}{2^{n}}-T(x_{1}),\ldots,\frac{f(2^{n}x _{k})}{2^{n}}-T(x_{k}) ) }^{k}(r)\geq\min_{i\in\mathbb{N}_{k}} \mu_{\frac{f(2^{n}x_{i})}{2^{n}}-T(x_{i})} \biggl( \frac{r}{k} \biggr) , $$

and

$$ \lim_{n\rightarrow\infty} \frac{f(2^{n}x_{k})}{2^{n}} = T(x_{k}). $$

We put $m= 0$ in (3.5), and we get

3.8 $$ \mu_{ ( f(x_{1})-\frac{f(2^{n}x_{1})}{2^{n}},\ldots ,f(x_{k})-\frac{f(2^{n}x _{k})}{2^{n}} ) }^{k} \biggl( \frac{t}{s} \biggr) \geq1-\frac{\alpha }{t}. $$

Then

3.9 $$\begin{aligned} &\mu_{f(x_{1})-T(x_{1}),\ldots,f(x_{k})-T(x_{k})}^{k} \biggl( \frac {2t}{s} \biggr) \\ & \quad \geq T_{M} \biggl( \mu_{ ( f(x_{1})-\frac{f(2^{n}x_{1})}{2^{n}},\ldots ,f(x_{k})-\frac{f(2^{n}x _{k})}{2^{n}} ) }^{k} \biggl( \frac{t}{s} \biggr) , \\ & \quad\quad \mu_{ ( \frac{f(2^{n}x_{1})}{2^{n}}-T(x_{1}),\ldots,\frac{f(2^{n}x _{k})}{2^{n}}-T(x_{k}) ) }^{k} \biggl( \frac{t}{s} \biggr) \biggr) \\ & \quad \geq 1-\frac{\alpha}{t} \end{aligned}$$

by (3.8) and when $n\rightarrow\infty$, which implies that (3.2).

Now, we show that *T* is additive. Let $x,y\in E$ and replace $x_{1},\ldots,x_{k}$ by $2^{n}x$, $y_{1},\ldots,y_{k}$ by $2^{n}y$, and *t* by $2^{n}t$ in (3.1). We get

$$ \mu_{ ( f(2^{n}\frac{x+y}{2})-\frac{f(2^{n}x)}{2}- \frac{f(2^{n}y)}{2},\ldots,f(2^{n}\frac{x+y}{2})-\frac{f(2^{n}x)}{2}- \frac{f(2^{n}y)}{2} ) }^{k} \biggl( \frac{2^{n}t}{s} \biggr) \geq 1- \frac{ \alpha}{2^{n}t}. $$

Using (NF4), we conclude that

3.10 $$ \mu_{\frac{f2^{n}(\frac{x+y}{2})}{2^{n}}-\frac{1}{2} \frac{f(2^{n}x)}{2^{n}}-\frac{1}{2}\frac{f(2^{n}y)}{2^{n}}} \biggl( \frac {t}{s} \biggr) \geq1- \frac{\alpha}{2^{n}t}. $$

On the other hand, we obtain that

3.11 $$\begin{aligned} \mu_{T(\frac{x+y}{2})-\frac{1}{2}T(x)-\frac{1}{2}T(y)} \biggl( \frac {4t}{s} \biggr) \geq& T_{M} \biggl( \mu_{T(\frac{x+y}{2})- \frac{f(2^{n}(\frac{x+y}{2}))}{2^{n}}} \biggl( \frac{t}{s} \biggr) , \\ & \mu_{\frac{T(x)}{2}-\frac{1}{2}\frac{f(2^{n}x)}{2^{n}}}\biggl(\frac{t}{s}\biggr), \\ & \mu_{\frac{T(y)}{2}-\frac{1}{2}\frac{f(2^{n}y)}{2^{n}}} \biggl( \frac {t}{s} \biggr) , \\ & \mu_{\frac{f(2^{n}(\frac{x+y}{2}))}{2^{n}}-\frac{1}{2} \frac{f(2^{n})}{2^{n}x}-\frac{1}{2}\frac{f(2^{n}y)}{2^{n}}} \biggl( \frac {t}{s} \biggr) \biggr) \\ \geq& 1-\frac{\alpha}{2^{n}} \end{aligned}$$

for each $x,y\in E$, $t,s\in\mathbb{N}$ with $(t,s)=1$. Utilizing again the density of $\mathbb{Q}$ in $\mathbb{R}$, we find that (3.11) remains true if $\frac{4t}{s}$ is substituted with a positive real number *r*.

Consequently,

$$ \mu_{T(\frac{x+y}{2})-\frac{1}{2}T(x)-\frac{1}{2}T(y)}(r)\geq1-\frac{ \alpha}{2^{n}} $$

for each $x,y\in E$ and $r\in\mathbb{R}$. Letting $n\rightarrow \infty$ reveals that *T* complies with Jensen, and using the fact that $T(0)=0$, we conclude that *T* is additive [27, Theorem 6].

It remains to show the uniqueness of *T*. Suppose that $T'$ is another additive mapping satisfying (3.2). Then, for each $t,s\in \mathbb{N}$, sufficiently large *n* in $\mathbb{N}$ and $x\in E$,

$$\begin{aligned} \mu_{T'(x)-T(x)} \biggl( \frac{t}{s} \biggr) &= \mu_{\frac{T'(2^{n}x)}{2^{n}}-\frac{T(2^{n}x)}{2^{n}}} \biggl( \frac {t}{s} \biggr) \\ &\geq T_{M} \biggl( \mu_{T'(2^{n}x)-f(2^{n}x)} \biggl( \frac {2^{n-1}t}{s} \biggr) , \mu_{T(2^{n}x)-f(2^{n}x)} \biggl( \frac{2^{n-1}t}{s} \biggr) \biggr) \\ &\geq1-\frac{\alpha}{2^{n-2}t} \\ &\geq1-\frac{\alpha}{2^{n-2}}. \end{aligned}$$

This inequality holds for each $r\in\mathbb{R}^{+}$ instead of $\frac{t}{s}$, too. Therefore, for each $r\in\mathbb{R}^{+}$, $n\in\mathbb{N}$, $\mu_{T'(x)-T(x)}(r)\geq1-\frac{\alpha}{2^{n-2}}$, letting $n\rightarrow\infty$, it follows that $T= T'$. □

## Conclusion

In this paper, we consider multi-Banach spaces, approximate by multiplicatives, and provide some controlled mappings, which are stable by control functions.
